# Novel and Efficient Method for Solid Phase Synthesis of Urea-Containing Peptides Targeting Prostate Specific Membrane Antigen (PSMA) in Comparison with Current Methods

**Published:** 2018

**Authors:** Mona Mosayebnia, Sedigheh Rezaeianpour, Pedram Rikhtechi, Zahra Hajimahdi, Davood Beiki, Farzad Kobarfard, Omid sabzevari, Mohsen Amini, Khosrou Abdi, Soraya Shahhosseini

**Affiliations:** a *Department of Radiopharmacy, Faculty of Pharmacy, Tehran University of Medical Sciences, Tehran, Iran.*; b *Phytochemistry Research Center, Shahid Behesti University of Medical Sciences, Tehran, Iran.*; c *Department of Pharmaceutical Chemistry and Radiopharmacy, School of Pharmacy, Shahid Behesti University of Medical Sciences, Tehran, Iran. *; d *Research Center for Nuclear Medicine, Tehran University of Medical Sciences, Tehran, Iran. *; e *Department of Medicinal Chemistry, and Drug Design and Development Research Center, Faculty of Pharmacy, Tehran University of Medical Sciences, Tehran, Iran. *; f *Protein Technology Research Center, Shahid Behesti University of Medical Sciences, Tehran, Iran*.; 1 M.M and S.R. contributed equally to this work.

**Keywords:** PSMA, Glutamate-Urea-Lysine, urea bond, solid phase, Isocyanate

## Abstract

The basic chemical structure of most prostate specific membrane antigen (PSMA) inhibitors which are now in pre-clinical and clinical studies is Glu-Ureido-based peptides. Synthesis of urea-based PSMA inhibitors includes two steps: 1- isocyanate intermediate formation and 2- urea bond formation. In current methods, isocyanate is formed in liquid phase and then reacts with amine existing in liquid phase or bound to solid phase for urea bond formation. In this study, we developed a new facile method for formation of both isocyanate and urea on solid phase under standard peptide coupling conditions. The solid phase-bound isocyanate served as intermediate to form urea bond. To monitor reaction progress qualitative test (Kaiser Test) and On-Bead FT-IR spectroscopy were used. The structure of Glutamate-Urea-Lysine (EUK) was confirmed using LC-Mass and 1H-NMR. This novel method successfully was applied to synthesize of another urea-based peptide containing a sequence of Glu-Urea-Lys (OMe)-GABA-Tyr-Tyr-GABA and the bifunctional linker hydrazinonicotinamide (HYNIC) as well.

## Introduction

Prostate cancer (PCa) is the most diagnosed cancer in adult men and the second cause of cancer deaths in North American and European men. It is estimated that one of the six men may be afflicted with PCa during their lives (1). There are such handful factors increasing the risk of PCa as: age, race, genetics, and lifestyle (2,3,12–21,4,22–24,5–11). The routine screening tests for diagnosis of PCa are prostate specific antigen (PSA) blood test and digital rectal examination (DRE) ^25-26^. In cases with high PSA level or any abnormal observations in DRE, prostate biopsy serves as an invasive method. CT-Scan, MRI, and prostate scintigraphy are main imaging techniques for detection of metastasis (27). There are some known biomarkers associated with PCa like (28) prostate stem cell antigen (PSCA) (29–31), prostate specific G-protein coupled receptors (PSGR), PSA (32–37) and PSMA (32–35). Of these markers, PSMA has gained more interest so as to early detection of PCa due to several reasons: 1) Over-expression on prostate cancer cells as membrane antigens 2) Broadly expression at all stages of prostate tumors 3) Up-regulation in androgen-insensitive and metastatic tumors 4) The absence on normal prostate cell surface with restricted expression in the brain, kidney, and small intestine. 5) Intrinsic enzymatic activity which makes it possible to design ligand-targeted drugs 6) Receptor-mediated Endocytosis of PSMA-targeted ligands leads to ligand retention into tumor cells.

Two distinct types of probes are used for targeting PSMA. The first groups were designed based on monoclonal antibodies (MAbs) which are not widely applied today (38). Low molecular weight pepidomimetics as PSMA inhibitors are the second attractive agents because of easy synthesis, high affinity, better tissue penetration, rapid clearance, and low immunogenicity (39). According to published studies, the active site of PSMA is of a funnel-like structure with an arginine patch that can be occupied by two main parts of PSMA inhibitors: glutamic acid or derivatives which ensures high affinity binding and zinc binding groups such as: Phosphonate, Sulfamide, Hydroxamate, Carbamate, and Urea moiety (40–42).

**Figure 1:**
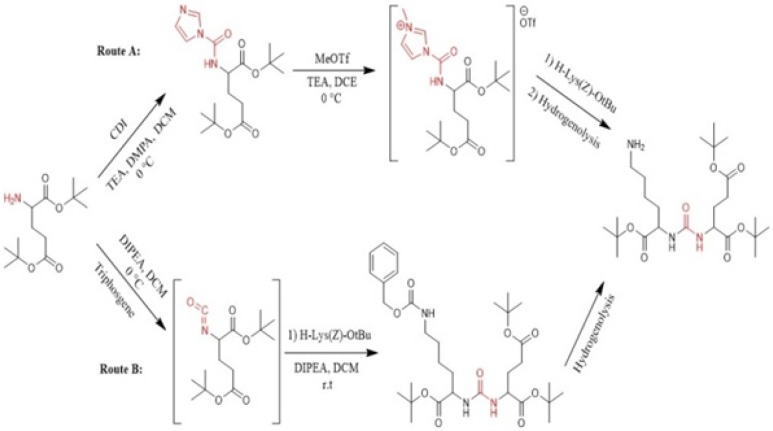
Synthesis of glu-urea-lys (EUK) in solution phase (45).

**Figure 2:**
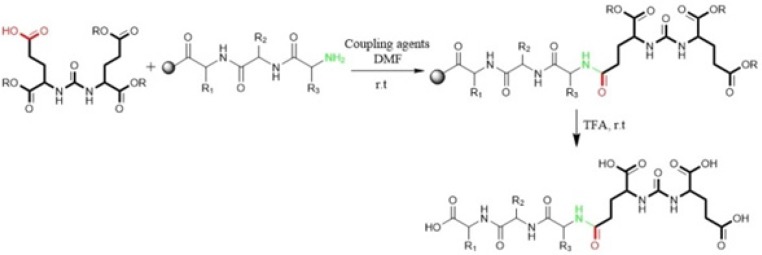
Synthesis of peptide containing glu-urea-glu (DUPA) motif for PSMA targeting. At first DUPA was formed by aqueous reaction between L-Glutamic acid di-tert-butyl ester and triphosgene. Then, it was conjugated to N-terminus of peptide residue. This method can’t be used for attaching the other pharmacophor of PSMA due to their absence of free Carboxylate (47).

**Figure 3:**
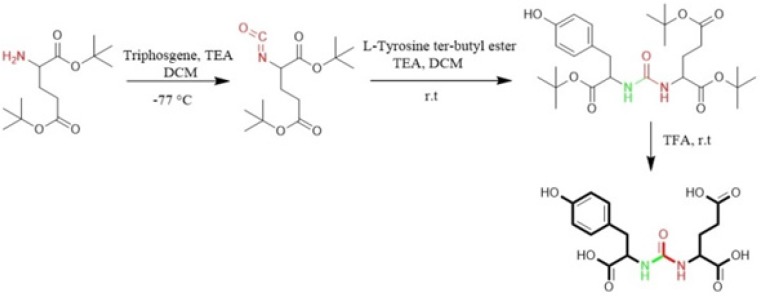
Liquid phase Synthesis of tyr-urea-glu (TUG) as a dipeptide PSMA inhibitor (50).

**Figure 4:**
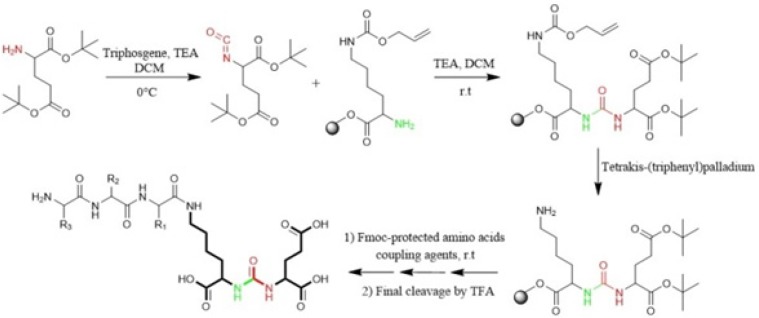
Synthesis of isocyanate intermediate under solution phase and then coupling with resin immobilized (2-chloro-tritylresin) ε-allyloxycarbonyl (Alloc) protected lysine in order to prepare glu-urea-lys (EUK)-containing peptides. During this method, there was no simple way to confirm the formation of isocyanate in solution phase (46).

**Figure 5:**
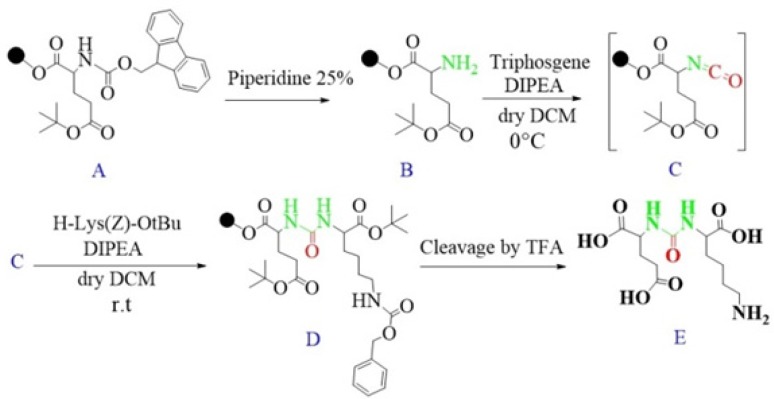
Schematic illustration of the synthesis route of glu-urea-lys (EUK) as a PSMA inhibitor.

**Figure 6:**
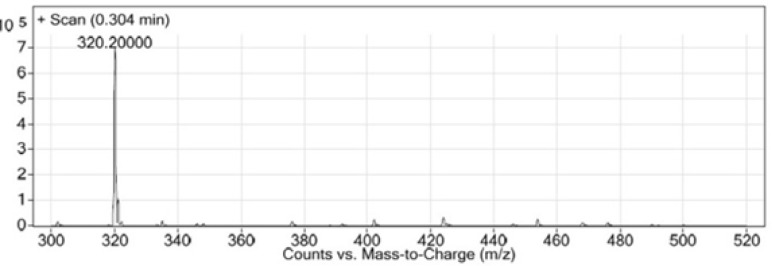
LC-MS chromatogram of synthesized Glu-Urea-Lys (EUK).

**Figure 7:**
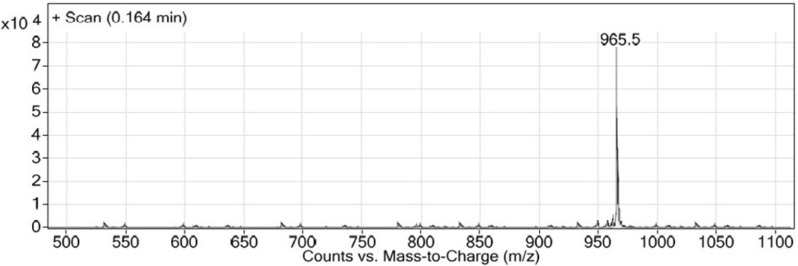
LC-MS chromatogram of Glu-Urea-Lys (OMe)-GABA-Tyr-Tyr-GABA-HYNIC.

**Figure 8:**
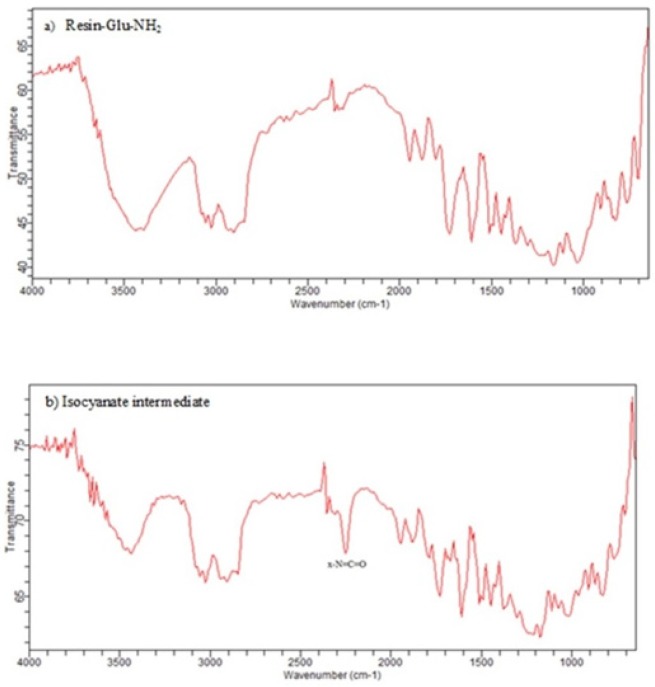
IR spectra of a) Deprotected glutamate bound to wang resin b) The corresponding isocyanate intermediate on wang resin. X-N=C=O stretch is seen in the 2270 cm^–1^ region of the IR (b) while it is absent in IR spectrum of resin-Glu-NH2 (a).

The basic chemical structure of most PSMA inhibitors which are now in pre-clinical and clinical studies is urea-based peptide. The pharmacophore, Glu-urea-R, specifically binds to PSMA and inhibits its activity. The Glu-Ureido-based inhibitors and its analogs are the most desirable probes since 2001. Relative simple synthesis and biological stability because of planar structure and neutral charge of ureido group might have made it ^44-45^. Four general urea containing pharmacophores have been introduced so far which are analogue of dipeptides: glutamate-urea-glutamate (EUE), glutamate-urea-cysteine (EUC), glutamate-urea-tyrosine (GUT), and glutamate-urea-lysine (EUK) (45). Unlike MAbs, peptide scaffolds as small molecules are synthesized easily by SPPS and more resistant in extreme radiolabeling conditions (temperature, pH). This is why they have been compared to the best candidate for designing radiopharmaceuticals (39).

Synthesis of urea-based PSMA inhibitors includes two steps: 1) isocyanate intermediate formation and 2) urea bond formation (43,45–50). The isocyanate intermediate is synthesized from the reaction of appropriate amino acid with tri/diphosgene in liquid phase under controlled conditions of temperature and pH. In the next step, the urea bond is formed from the reaction of amino acid free amine with isocyanate. Overall, these current methods involve conjugation of free amine whether in liquid phase ^44,46,51-52^ or bound to solid phase (46) with isocyanate formed in solution phase before. 

In recent years, a variety of radioligands targeting prostate-specific membrane antigen (PSMA) have been clinically developed as a new class of radiopharmaceuticals for prostate cancer. Sigurdsson *et al*. in 1999 prepared isocyanate from aliphatic amines using trichloromethyl chloroformate (diphosgene) at 0 °C in presence of the non-nucleophilic base (51). Kozikowski *et al. *in 2001 reported the synthesis of urea-based glutamate carboxypeptidase II (NAALADase) inhibitors in which triphosgene has been used for carbonylation of amine in liquid phase at -78 º C. Afterwards, the corresponding intermediate reacted with free amine of the other amino acid to form urea moiety (43). Hiller *et al*. in 2009 synthesized a series of Glu-urea-X heterodimers as PSMA inhibitors, which X is a derivatized lysine (Lys). The compounds were radioiodinated and theirs affinity to prostate cancer cells were determined. The urea linkage was synthesized in liquid phase utilizing two routes: acylimidazole intermediate afforded by Carbonyldiimidazole (CDI) (route A) and isocyanate intermediate prepared by Triphosgene (route B) (Figure 1) (45).

Kularatne *et al* in 2009 prepared a series of (Glu-urea-Glu)-based PSMA inhibitors. At first, The Glu-urea-Glu pharmacophore (EUE) was synthesized through isocyanate intermediate formation in liquid phase using triphosgene under controlled conditions. Then, one of the carboxylate groups in the Glu-urea-Glu was de-protected via catalytic hydrogenation and subsequently conjugated to N-terminal of resin-bound peptide to furnish final peptide. Actually, in this procedure both solid phase and solution phase peptide synthesis techniques have been applied (Figure 2) (47).

Al-momani *et al.* in 2012 only took advantage of solution phase peptide synthesis method to prepare Glu-Urea-Tyr. The reaction of triphosgene in CH_2_Cl_2_ with bis (tert-butyl)-L-glutamate.HCl using triethylamine as a base, at -77 °C under argon, resulted in isocyanate intermediate. In next step, L-Tyrosine tert-butyl ester were activated by triethyamine and conjugated with isocyanate to afford urea linkage (Figure 3) (50).

Eder *et al* in 2012 synthesized Glu-NH-CO-NH-Lys (Ahx)-HBED-CC. To form urea bond, the isocyanate of glutamyl moiety (Glu-N = C = O) was synthesized in liquid phase and then reacted with free α-amino group of resin-bound ε-allyloxycarbonyl (Alloc) protected lysine. So, the peptide chain elongation was completed in solid phase (Figure. 4) (46).

Zhang *et al*. in 2016 used similar liquid phase procedures using triphosgene under controlled conditions (temperature, pH, anhydrous and N_2_) for synthesis of four analogs of Glu-Urea-R. They also determined the affinities of synthesized compounds to PSMA (49). 

In the present study, we introduce a rapid method for solid phase synthesis of isocyanate and urea moiety using peptide coupling reagents.

## Experimental

*Materials*

*Reagents: *All protected amino acids and 2-chlorotrityl chloride (2-CTC) resin were of analytical grade from Novabiochem (Merck, Darmstadt, Germany). Triphosgene was purchased from Merck Co. (Germany), TBTU (O-(Benzotriazol-1-yl)-N, N, N′, N′-tetramethyluronium tetrafluoroborate), DIPEA (Diisopropylethylamine), TFA (Trifluoroaceticacid), TIS (Triisopropylsilan) and solvents such as: DCM (Dichloromethane), MeOH (Methanol), and DMF (Dimethylformamide) were obtained from Sigma-Aldrich (USA) and used as received. High-resolution LC-MS Triple Quad 6410 (Agilent) with a series 1200 HPLC column (Japan) (C-18, 250 Å~4.6 mm, 5 µm) were used for recording Mass Spectra of peptide. Mobile phase: A: H_2_O + 0.1 % TFA, B: Acetonitrile, flow rate: 1 mL/min, volume of injection 20, total run time: 40 min. Bruker AVANCE III HG 44 MHz NMR was used for ^1^H-NMR spectra of peptide. 

*Synthesis method*

Synthesis of Glu-Urea-Lys (EUK): The EUK was synthesized using a standard Fmoc strategy by 2-chlorotrityl chloride (2-CTC) resin as a solid phase. Briefly, Fmoc-Glu (OtBu)-OH (2 mmol) was attached to 2-CTC resin (1.0 g) using DIPEA (8 mmol) in anhydrous DCM/DMF (10 mL, 1:1) for 2 h at room temperature (Figure 5, A). 

Then, the reaction mixture was filtered. The remaining trityl chloride groups of the resin were capped by 24 mL solution of DCM/MeOH/DIPEA (17:2:1) within 30 min. The resin was washed with DMF (3 × 5 mL) and DCM (1 × 5 mL) and filtered off. The Fmoc group was removed using 25 % piperidine/DMF (13 mL) for 30 min (Figure 5, B).

To form isocyanate intermediate, in a round bottom flask, DIPEA (4.5 mmol) and triphosgene (0.6 mmol) in 10 mL of dry DCM was added to resin (resin-Glu-NH_2_). The mixture was stirred for 6 h at 0 ºC in sealed conditions (Figure 5, C). 

After this period, the mixture was moved to the reaction vessel, filtered, and washed with dry DCM (3 × 5 mL). The completeness of the reaction was checked by Kaiser Test. In the next step, H-Lys (Z)-OtBu (2 mmol) dissolved in 5 mL dry DCM and DIPEA (4.5 mmol) were added into reaction vessel and agitated at ambient temperature overnight. Afterwards, the solution was filtered and resin was washed 3 times using 5 mL of DCM and 100 µL of DIPEA to remove unreacted Lysine. The end-point of lysine attachment to isocyanate was indicated by positive Kaiser Test (Figure 5, D). 

The peptide was cleaved from surface of the resin using 100 mL of 1% TFA in DCM and neutralized with 50 mL 4% pyridine in MeOH. The solvents were removed under reduced pressure in a rotary evaporator, and the peptide was precipitated in distilled water. All side-chain protecting groups of the peptide sequence (OtBu) were deprotected by treatment with a cleavage cocktail TFA/TIS/H_2_O (95:2.5:2.5). According to Albert Isidro-Llobet and *et al* (2009), the Z group was partially removed in presence of cocktail TFA/scavenger as well (52) (Figure 5, E).

After removing of the solvents, the peptide was precipitated into excess cold diethyl ether and finally valuable product was isolated by preparative HPLC. As a marker for free amine group, Kaiser Reagent (consists of 2 solutions, solution A: 80 g of Phenol in 20 mL of Ethanol, solution B: 2 mL of 2 M KCN in 100 mL of pyridine) was used, which in presence of free amine, resin seeds turn into dark blue. Chloranil test is also used to check it by 5 drops of both 2% Chloranil solution and 2% Acetaldehyde solution which in the case of free amines, resin seeds appear as dark-blue. The schematic diagram for synthesis of EUK is illustrated in Figure 5. The identity of peptide was confirmed by LC-MS (Figure 6).

Synthesis of new urea-based peptide: To synthesize long peptide being of O-methylated derivative of EUK, H-Lys (Fmoc)-OMe was used instead of Z-protected Lysine. Firstly, the resin-bound Glu-Urea-Lys (OMe) was prepared as described above. After Fmoc deprotection of Lysine, a solution of the next amino acid (2 mmol), TBTU (2 mmol), and DIPEA (2.5 mmol) in 6 mL of DMF was added to assemble the third amino acid on resin (1 h at room temperature). The deprotection, coupling, and washing cycles were repeated until assembly of Fmoc-protected GABA-Tyr-Tyr-GABA-HYNIC peptide was complete. The bifunctional hydrazinonicotinamide (HYNIC) moiety was used so as to radiolabel of this new peptide scaffold in future studies. LC-Mass chromatogram was shown in Figure 7. 

## Results

In this study, Glu-Urea-Lyz (EUK) was synthesized on solid phase under peptide coupling conditions. The LCMS (Figure 6) and ^1^H NMR spectroscopy were used to confirm the identity of synthesized compound.

LC-MS (m/z): (M+H)^+^. Calculate for C_12_H_21_N_3_O_7_, 319; found, 320.

Glu-Urea-Lys: white powder, ^1^H NMR (400 MHz, CDCl_3_): δ 0.87 (m, 1H), 1.09-1.33 (m, 4H), 1.69-1.92 (m, 4H), 1.92 (m, 1H), 2.24 (m, 2H), 2.77 (m, 3H), 4.08 (m, 4H), 5.00 (s, 1H, NH), 6.35 (s, 2H, NH), 7.35-7.68 (m, 5H). 

This novel method was used for synthesizing another urea-containing peptide, Glu-Urea-Lys(OMe)-GABA-Tyr-Tyr-GABA-HYNIC: white powder. LC-MS (m/z): (M+H)^+^. Calculate for C_45_H_60_N_10_O_14_, 964.5; found, 965.5 (Figure 7).

## Discussion

Regarding an increasing trend in prevalence of PCa in men over 40 years and its high mortality rate, researches are taking place for early detection of prostate tumors (48). There are a few markers to be particularly found on prostate cancer cells. PSMA is a valuable specific antigen on the surface of tumor cells with enzymatic roles. Analysis of crystal structure of PSMA and active site of enzyme has resulted in design and synthesis of several classes of PSMA inhibitors including monoclonal antibodies, aptamers, and small molecules such as peptides. The more attractive targeted agents are urea-based dipeptide scaffolds in which two amino acids are joined through α-amino groups by urea linkage. Based on structure activity studies of amino acids and potency of PSMA inhibitors, Glu-urea-R is an appropriate pharmacophore of PSMA inhibitors. A few urea-based small molecules for targeting PSMA are in pre-clinical and clinical studies. Glu-urea-R is synthesized via formation of the intermediate isocyanate which is formed in liquid phase. The traditional synthetic pathway to intermediate isocyanate formation is using carbonyl insertion reagents such as tri/diphosgene in liquid phase under controlled conditions of temperature and pH. 

In this study, for the first time, we generated isocyanate intermediate on resin (Resin-Glu-N=C=O) which has considerable advantages over liquid phase strategy. The Fmoc-amino acid (tert-butyl-protected Glutamic acid) was loaded onto the wang resin and the Fmoc group removed. The solid phase-bound glutamate was then reacted with triphosgene in the presence of DIPEA as a base. The corresponding isocyanate on solid phase was not isolated and directly reacted with H-Lys (Z)-OtBu to generate the urea linkage (Resin-Glu-NH-CO-NH-Lys). On-Bead Fourier transform infrared (FT-IR, Agilent) spectroscopy of the intermediate isocyanate showed one major band at approximately 2250-2270 cm^-1 ^after reaction between the solid-phase bound amine and triphosgene. This band was absent in IR spectrum of deprotected glutamate bounded to the resin (Figure 8). The LCMS (Figure 6) and ^1^H NMR spectroscopy were used to confirm the identity of the synthesized compound.

There are some positive aspects of using on-resin generation of isocyanate versus isocyanate formation in liquid phase like ease of synthesis and reaction progress monitoring by qualitative test. Di-Glutamate and HCl can be formed during isocyanate formation step in liquid phase though. HCl will be neutralized by DIPEA present in the reaction mixture but there is no simple way to remove di-glutamate impurity from solution phase. In solid phase, isocyanate is prepared on-resin which makes the formation of di-glutamate impurity decrease. Furthermore, this impurity no longer participates in the next coupling reactions due to absence of Fmoc-protected amine in its structure. The resin-bound isocyanates are separated from unreacted triphosgene and glutamate after washing and filtration steps resulting in high purity of final product. 

The isocyanate formation on resin is also monitored via qualitative Chloranil or Kaiser Test within a few minutes. The light-yellow color of beads indicates the absence of free primary amino groups (-NH_2_) which have converted into isocyanate groups (-N=C=O). In addition to, FTIR spectroscopy a single resin bead can be used to monitor of the solid-phase reaction.

Two approaches have been adopted to convert isocyanate into urea. In the first approach, amino acid reacts with isocyanate in liquid phase to form urea linkage. In the second one, excess amounts of isocyanate in solution phase are reacted with amino acid bound to resin (46). In these approaches, the structure of isocyanate is not confirmed before the next step, while our procedure enables us to monitor isocyanate formation via Chloranil or Kaiser Test within a few minutes. 

To afford longer peptide sequence, some studies have employed solid-phase for conjugation of prepared urea-containing pharmacophore to the N-terminal of peptide bound to resin (47). The free carboxylic acid group of amino acid is required for this strategy. 

Herein, we introduce an applicable solid-phase synthesis protocol that allows simple synthesis of Glu-Ureido–Based long peptides with high purity. The one-pot reaction between the amine, triphosgene and DIPEA gives isocyanate. The isocyanate on solid phase is not removed and reacts with amine for direct formation of urea bond. The method is similar to peptide synthesis on solid phase and can be easily adopted for solid phase synthesis (SPS) of urea-based peptides. 

## Conclusion

In the present study, an efficient and versatile synthesis of Glu-Ureido–Based PSMA inhibitors using solid phase protocol was developed. In this method, the isocyanate and subsequent urea formation take place under standard peptide coupling conditions using triphosgene as an amino acid. Urea-containing peptides can be easily cleaved from resin at the end of the synthesis using acidic conditions. The aim of this study is to generate isocyanate intermediate directly on resin which is well suited for peptide chain elongation with high purity. 
